# Surface Free Energy and Composition Changes and Ob Cellular Response to CHX-, PVPI-, and ClO_2_-Treated Titanium Implant Materials

**DOI:** 10.3390/jfb13040202

**Published:** 2022-10-25

**Authors:** Roland Masa, István Pelsőczi-Kovács, Zoltán Aigner, Albert Oszkó, Kinga Turzó, Krisztina Ungvári

**Affiliations:** 1 Department of Oral Biology and Experimental Dental Research, Faculty of Dentistry, University of Szeged, Tisza Lajos krt. 64., H-6720 Szeged, Hungary; 2 Department of Prosthodontics, Faculty of Dentistry, University of Szeged, Tisza Lajos krt. 64., H-6720 Szeged, Hungary; 3 Institute of Pharmaceutical Technology and Regulatory Affairs, Faculty of Pharmacy, University of Szeged, Zrínyi u. 9., H-6720 Szeged, Hungary; 4 ELI-HU Non-Profit Ltd., Wolfgang Sandner u. 3., H-6728 Szeged, Hungary; 5 Dental School, Medical Faculty, University of Pécs, Tüzér u. 1, H-7623 Pécs, Hungary

**Keywords:** antibacterial agents, decontamination, dental implants, titanium, peri-implant infections

## Abstract

The study evaluated the interaction of a titanium dental implant surface with three different antibacterial solutions: chlorhexidine, povidone-iodine, and chlorine dioxide. Implant surface decontamination is greatly challenging modern implant dentistry. Alongside mechanical cleaning, different antibacterial agents are widely used, though these could alter implant surface properties. Commercially pure (CP) grade 4 titanium (Ti) discs were treated with three different chemical agents (chlorhexidine 0.2% (CHX), povidone-iodine 10% (PVPI), chlorine dioxide 0.12% (ClO_2_)) for 5 min. Contact angle measurements, X-ray photoelectron spectroscopy (XPS) analysis, and cell culture studies were performed. Attachment and proliferation of primary human osteoblast cells were investigated via MTT (dimethylthiazol–diphenyl tetrazolium bromide), alamarBlue, LDH (lactate dehydrogenase), and fluorescent assays. Contact angle measurements showed that PVPI-treated samples (Θ = 24.9 ± 4.1) gave no difference compared with controls (Θ = 24.6 ± 5.4), while CHX (Θ = 47.2 ± 4.1) and ClO_2_ (Θ = 39.2 ± 9.8) treatments presented significantly higher Θ values. All samples remained in the hydrophilic region. XPS analysis revealed typical surface elements of CP grade 4 titanium (Ti, O, and C). Both MTT and alamarBlue cell viability assays showed similarity between treated and untreated control groups. The LDH test revealed no significant difference, and fluorescent staining confirmed these results. Although there was a difference in surface wettability, a high proliferation rate was observed in all treated groups. The in vitro study proved that CHX, PVPI, and ClO_2_ are proper candidates as dental implant decontamination agents.

## 1. Introduction

Commercially pure (CP 1–4) Ti and its alloys represent the standard material of dental implants since they have excellent mechanical and physicochemical properties [1,2]. However, the ideal dental implant surface, which both inhibits bacterial colonization and promotes host cell attachment, has not yet been found [3].

The number of dental implant placements increases every year [4], and the prevalence of implant failures escalates in parallel. The major causes of these failures are infections and overloading of the implant-based denture. The main goal is prevention, but in the case of infections, implant surface decontamination is essential. In the regenerative therapy of peri-implantitis, in addition to resolving the infection, the goal is to promote bone regeneration around the implant. Inflammation-free conditions that facilitate bone regeneration and re-osseointegration are important, and osteoblasts are involved in this process [5].

Several chemical surface decontaminants have been proposed as a supplemental therapy in the nonsurgical or surgical treatment of peri-implantitis. Chlorhexidine (CHX), citric acid (CA), hydrogen peroxide (H_2_O_2_), ethylenediaminetetraacetic acid (EDTA), chloramine-T, and local antibiotics are commonly used cleaning components [6,7,8].

Chlorhexidine is one of the most widely applied and investigated antiseptic materials in dental practice [9]. CHX attachment to saliva-coated surfaces promotes long-term antibacterial effects and the inhibition of plaque formation [10,11]. Different forms (mouthwash, spray, gel) and concentrations (0.02–2%) of CHX are available [9]. Bacterial resistance could not develop against CHX even in cases of long-term use [12].

Povidone-iodine has a remarkably broad antibacterial spectrum, with a low risk of adverse reactions and bacterial resistance [13]. The main advantage of this iodophor is having all the iodine in a complex form; therefore, its irritating and staining effects are negligible [14]. Allergic contact dermatitis to PVPI in the case of short contact is rare (0.4%), yet its use is not recommended for patients with a pre-existing iodine allergy [15]. Adjunctive use of PVPI in the therapy of periodontitis [16] and peri-implantitis [6,17] has been reported by several authors. On the other hand, cellular toxicity of epithelial cells exposed to PVPI has been reported [18].

Chlorine dioxide (ClO_2_) antiseptics could be a promising alternative to CHX, since no significant difference has been found between ClO_2_ and CHX mouthwashes in reducing the bacterial load, nor gingival and plaque indexes in recent studies [19,20]. Other possible applications of this agent, such as reducing oral malodor [21] and disinfecting dental instruments [22], have also been reported. A high-purity stabilized form of ClO_2_ as an antibacterial agent has recently been introduced to the market. This antimicrobial compound requires only a short time to kill bacteria, and its penetration into human tissues is below 0.1 mm, which makes it safe to use [23]. The antibacterial effect of ClO_2_ is based on its reaction with four crucial amino acids (cysteine, tyrosine, tryptophan, and methionine) and inorganic ions. Its biofilm-dissolving ability was found to be excellent [24].

Besides surface topography and roughness, chemical composition and surface free energy are both of key importance regarding host–biomaterial interactions [25] for an optimal biointegration. Reduced initial protein adsorption and altered cellular response can be the undesired consequences of poor surface wettability [26]. The goal would be to preserve the original hydrophilicity of the implant surface even after the elimination of severe inflammation.

Specific decontaminating agent residues could alter implant surface properties and osteoblast responses [27]. Our aim was to evaluate the interaction of a titanium dental implant surface with three different antibacterial solutions: CHX (without any staining effect [28]) and two less investigated compounds, povidone-iodine and chlorine dioxide. The possibility of changes in the physical features and chemical composition of the discs was investigated.

## 2. Materials and Methods

### 2.1. Preparation of Ti Samples

Ti sample discs (1.5 mm thick and 9 mm in diameter) were made of commercially pure (CP4) titanium rods (Denti System^®^ Ltd., Szentes, Hungary). In this in vitro study, sandblasted and acid-etched discs were used. The surface roughness of the samples was measured previously by our research group (R_a_ = 544 ± 47 nm) by AFM (PSIA XE-100 instrument, PSIA Inc., Korea) [29].

The samples were treated with three different chemical agents: chlorhexidine digluconate (Curasept ADS 220, 0.2%, Switzerland), povidone-iodine (Betadine, 10%, Switzerland), and chlorine dioxide (Solumium Dental, 0.12%, Hungary), each for 5 min. After the treatment, the discs were rinsed in ultrapure water three times. Control discs were rinsed exclusively with ultrapure water.

### 2.2. Contact Angle (CA) Measurements

CA measurements were performed by a video-camera-based instrument (OCA 20, DataPhysics, Germany) with purified water (PW, pharmaceutical grade, Ph.Hg. VIII. European Pharmacopoeia 9.0, European Directorate for the Quality of Medicines and HealthCare, France) and diiodomethane (MI, Merck KGaA, Germany) drops. One measurement was performed on each disc. The volume of the drops was 10 µL with a constant distance from the surface of 10 mm. This sessile drop method was performed on 6 chemical-agent-treated Ti discs per group. Wettability assays were evaluated with the SCA 20 and 21 software (DataPhysics Instruments GmbH, Germany). Surface free energy (SFE) (*γ* (mJ/m^2^)) was determined according to the Owen–Wendt–Rabel–Kaelble (OWRK) method—commonly used to evaluate Ti implant surfaces [30,31].

### 2.3. X-ray Photoelectron Spectroscopy

The chemical compositions of the control and treated Ti surfaces were studied using XPS–LEIS. The photoelectrons were generated by Al Kα primary radiation (*hν* = 1486.6 eV) and examined with a hemispherical electron energy analyzer (PHOIBOS 150 MCD 9, SPECS, Germany). The X-ray gun was operated at 150 W (12 kV, 12.5 mA). The binding energies normalized with respect to the position of the C 1s peak of adventitious carbon, which was considered to be 285.1 eV. Wide-range scans and high-resolution narrow scans of the Ti 2p, O 1s, and C 1s characteristic peaks were recorded [32].

### 2.4. Cell Culture Studies

Cell culture experiments with primary oral human osteoblast cells were carried out in three replicates. Bone chips were obtained from three healthy adult patients who had undergone routine dentoalveolar surgery. Osteoblast cells were isolated from tiny bone fragments removed during surgical extraction of molars, with the written consent of the patients. The study protocol filled every requirement of the Declaration of Helsinki in all respects; moreover, it was approved by the Regional Research Ethics Committee for Medical Research at the University of Szeged (188/2013).

The osteoblast cells were separated from bone chips during enzymatic digestion [33]. The medium consisted of Dulbecco’s Modified Essential Medium (DMEM) (Corning USA). Primary osteoblast cells were passaged at least three times before the experiments.

Cells were pipetted onto the Ti discs at a density of 10^4^ cells/well and cultured in sensitive 48-well plates. Cell culture plate wells (henceforth plate) were used without discs as a positive control. The attachment of the cells was determined after 24 h, and the proliferation rate was measured after 72 h. The experiments were repeated three times, and four samples were used for each assay. Cellular viability was assessed through common colorimetric assays (MTT, alamarBlue, LDH). Fluorescent staining as a qualitative method was also applied to examine osteoblast morphology.

#### 2.4.1. MTT Assay

The MTT reaction determines the mitochondrial activity of living cells via the reduction of tetrazolium salt. After 24 and 72 h, the culture medium was removed and replaced with 1 mg/mL MTT (Sigma-Aldrich GmbH, Germany) solution in DMEM. After 4 h, this medium was removed, following which the formazan crystals were solubilized with 200 µL dimethyl sulfoxide (DMSO) (Corning, USA). The optical density was determined at 570 nm (OD_570_) using a Multiskan GO spectrophotometer (Thermo Fisher Scientific, USA).

#### 2.4.2. AlamarBlue Assay

AlamarBlue^®^ (AB, G-Biosciences, USA) is a water-soluble indicator dye used for quantitative measurement of the proliferation of mammalian cells’ digestion [34]. The dye contains an oxidation–reduction indicator (resazurin), which can change its color due to chemical reduction resulting from cell growth. According to the manufacturer’s recommendations, the culture medium was supplemented with 10% of AB after 24 and 72 h and incubated for 3.5 h. The optical density was measured at 570 and 600 nm with a Multiskan GO spectrophotometer (Thermo Fisher Scientific, USA).

#### 2.4.3. LDH Activity

LDH (lactate dehydrogenase) is a stable cytoplasmic enzyme that is present in almost every cell. The release of this enzyme in the culture supernatant is directly proportional to the number of damaged cells and can be detected photometrically using standard reagents [35]. An LDH cytotoxicity kit (Takara Bio Inc., Kusatsu, Japan) was used according to the manufacturer’s instructions. Osteoblasts were cultured and then lysed with 1% Triton X (Roth Industries, Dautphetal, Germany) on control Ti discs, which served as a positive control. The optical density was measured at 492 and 620 nm with a Multiskan GO spectrophotometer. Cytotoxicity (%) of the treated discs was determined by OD values of control discs (low control) and Triton X–treated discs (high control).

#### 2.4.4. Visualization with Fluorescent Microscopy

Cell nuclei were labeled with bisbenzimide Hoechst 33,342 blue dye (Merck Millipore, Germany) and the cytoskeleton with the red phalloidin–tetramethylrhodamine B isothiocyanate (TRITC-phalloidin, Sigma-Aldrich GmbH, Germany). Images were taken with a Nikon Eclipse 80i fluorescent microscope (Nikon Corporation, Minato-Ku, Japan) at a magnification of ×100. In each case, two images were taken using two different filters (DAPI, ex. 320, 520 nm; TRITC, ex. 510–560 nm) with the position of the samples unaltered. To produce composite pictures, the ImageJ 1.47 v software (National Institutes of Health, Bethesda, MD, USA) was used.

### 2.5. Statistical Analyses

The means ± the standard deviations (SD) were calculated for the contact angle values (Θ (°)), and the chemical-agent-treated groups were compared with the control group via unpaired Student *t*-test. The means ± the standard error of the mean (SEM) were calculated for each OD values yielded by optical densitometry. After normality testing, the data were compared via nonparametric Kruskal–Wallis test (SPSS 21, Chicago, IL, USA). The level of significance was set at *p =* 0.05.

## 3. Results

### 3.1. CA Measurements

**Figure 1:**
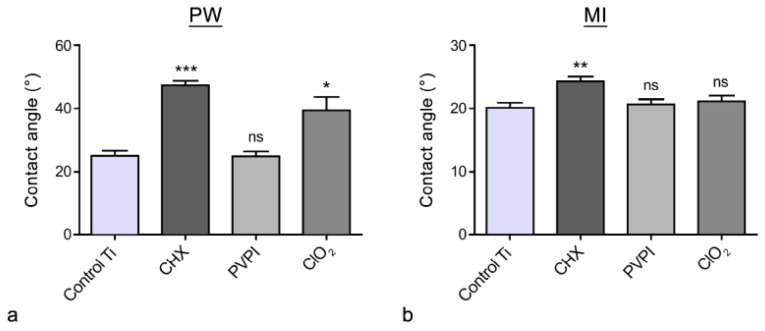
Surface wettability of the different chemical-agent-treated samples determined with PW (**a**) and MI (**b**) drops. Data are presented as mean ± SD. ns = not significant; asterisks denote significant differences (* *p* < 0.05, ** *p* < 0.01 and *** *p* < 0.001).

The contact angle values of the samples are presented in Figure 1. In the case of the PW drops, the control titanium samples showed an average Θ ± SD of 24.6 ± 5.4. No significant difference was found in the PVPI-treated group (Θ *=* 24.9 ± 4.1) compared with the control discs. ClO_2_ (Θ *=* 39.2 ± 9.8)- and CHX (Θ *=* 47.2 ± 4.1)-treated discs showed significantly higher (*p*_ClO2_ = 0.012; *p*_CHX_ < 0.0001) contact angle values compared with the control group.

The other group was tested with MI drops. The following values were measured: Θ_control Ti_ = 20.1 ± 2.1; Θ_PVPI_ = 20.6 ± 2; Θ_ClO2_ = 21.1 ± 2.3; Θ_CHX_ = 24.3 ± 1.7. This measurement indicated smaller differences among groups; only the CHX-treated group had significantly higher Θ (*p* = 0.003) compared with the control.

Based on the OWRK method, the SFE (γ) and its disperse (γ^d^) and polar (γ^p^) components were determined with the SCA software (DataPhysics Instruments GmbH, Germany). The results are presented in Table 1. From the three antiseptic solutions, only PVPI treatment could preserve similar SFE (γ = 70.7 mJ/m^2^) to the control surface (γ = 70.9 mJ/m^2^). Significant differences were observed for γ after CHX and ClO_2_ treatment, with γ = 59.5 mJ/m^2^ and γ = 64.1 mJ/m^2^, respectively. This resulted in a decrease in the polar component of the SFE (the disperse component remained almost identical).

### 3.2. XPS Results

**Figure 2:**
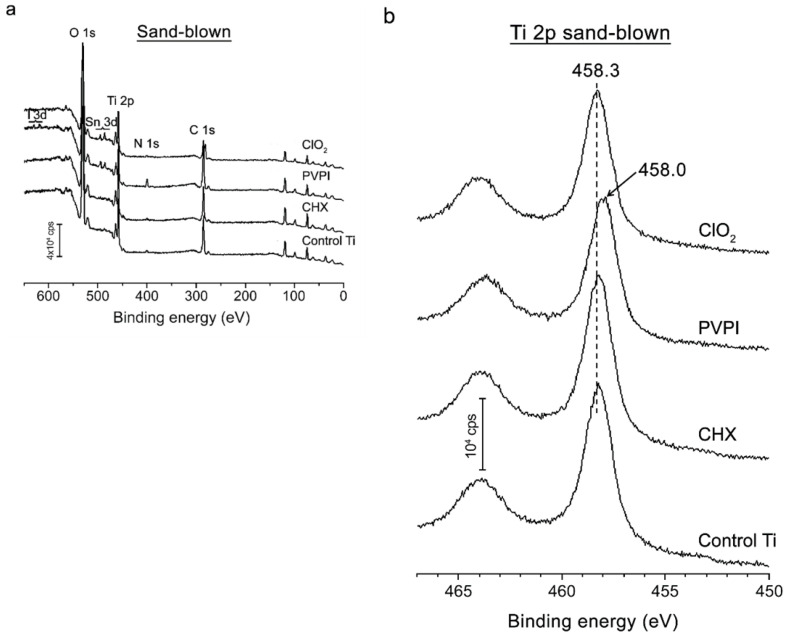
XPS spectra (**a**) of the control and the treated titanium discs. High-resolution XPS spectra (**b**) showing Ti 2p lines of untreated and treated titanium samples.

The XPS spectra illustrated in Figure 2a confirmed the presence of Ti, O, and C on both the untreated and treated Ti samples. These elements are typically observed on investigated surfaces with the positions and intensity of the O 1s and Ti 2p peaks proving the presence of an intact TiO_2_ layer [36], with characteristic peaks at 464.5 eV (Ti 2p_1/2_) and 458.6 eV (Ti 2p_3/2_). The C 1s signal (peak at ~290 eV) indicates the presence of carbonaceous contamination due to carbon-containing molecules adsorbed later, on air-exposed surfaces [37,38].

Representative high-resolution Ti 2p spectra of the investigated Ti samples are shown in Figure 2b. A shoulder with a peak around 462 eV can be observed, which can be attributed to a metallic or TiN compound. The presence of a metallic peak in the surface spectra is due to the fact that the surface oxide is thin enough to allow photoelectrons from the metal just beneath the metal–oxide interface to escape through the oxide [39]. Trace amounts of N could also be detected in the samples treated with PVPI or CHX with peaks at ~453.5 and ~400 eV as a result of the process of sample fabrication. Based on the XPS spectra, it can be generally concluded that the treatments did not significantly alter the surface chemistry [40].

### 3.3. Cell Culture Studies

#### 3.3.1. MTT Assay

**Figure 3:**
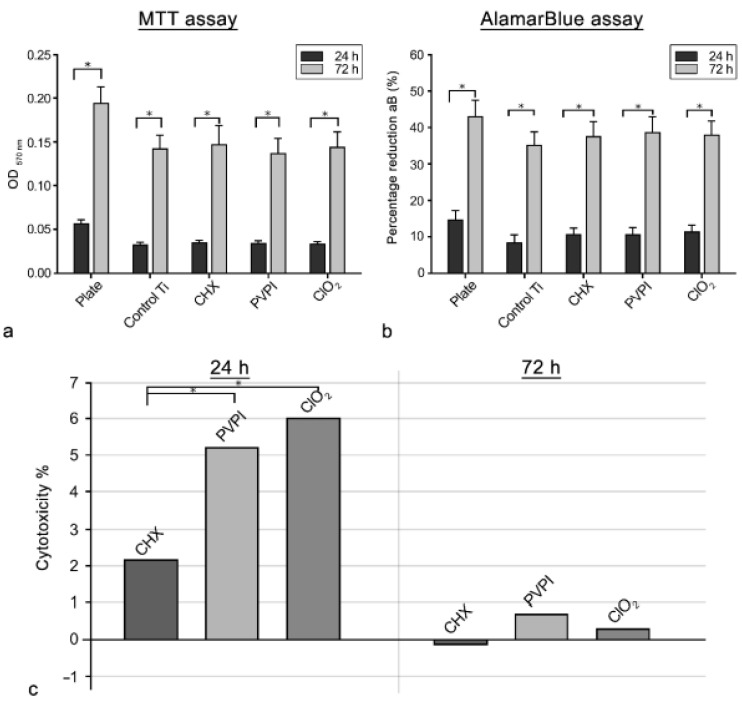
Attachment (24 h) and proliferation (72 h) values of osteoblast cells incubated with MTT (**a**) on the control (plate and uncovered Ti) and chemical-agent-treated discs. Data are presented as mean ± SEM. Percentage reduction of alamarBlue (**b**) on the control (plate and uncovered Ti) and chemical-agent-treated discs. Data are presented as mean % ± SEM. Cytotoxicity was measured by the release of lactate dehydrogenase from the cultivated osteoblast cells (**c**). The bar graphs illustrate the % difference in cell death of the treated discs compared with the control discs. Data are presented as mean %. Asterisks denote significant differences (* *p* < 0.05).

The mitochondrial activity of the osteoblast cells, in parallel with the living cell number, is shown on the bar graph of Figure 3a. Cellular attachment of the osteoblasts was significantly higher on the control plate (*p* < 0.001) than on the tested discs, while no significant difference was found between the control disc and the chemical-agent-treated discs.

After 72 h (proliferation), similar OD_540_ values were recorded on all of the investigated groups, with no statistical difference. The contaminating effects of the different antibacterial agents were negligible with respect to cellular response based on the MTT test.

From a different perspective, a considerable increase was observed in cell number in each of the examined groups. Moreover, the rate of proliferation (4.5×) was even higher on the treated surfaces than on the control plate (3.8×). This significant rate of proliferation (*p* < 0.001) demonstrates the viability of the investigated primary osteoblast cells.

#### 3.3.2. AlamarBlue

The viability of osteoblast cells was estimated through the percentage reduction of the resazurin dye. The results are shown on the bar graphs in Figure 3b.

No significant difference was observed between the control and treated groups, after either 24 or 72 h. However, a significant difference (*p* = 0.021) was observed on the percentage reduction in alamarBlue on all samples when comparing the 24 and 72 h values. These results support the results of the MTT assay.

#### 3.3.3. LDH Assay

A common cytotoxicity study was also carried out in order to determine the rate of damaged cells in the treated and control groups. Cytotoxicity % values of treated discs are illustrated in the bar graph in Figure 3c. The CHX group (2%) was found to be the least cytotoxic compared with other groups (<5%) after 24 h. There was no notable difference in the number of damaged cells in the treated Ti samples (~0% cytotoxicity) after 72 h compared with controls. Low release of LDH in the treated groups means that the titanium surfaces remained highly cytocompatible after treatment with the antibacterial solutions.

#### 3.3.4. Visualization with Fluorescent Microscopy

**Figure 4:**
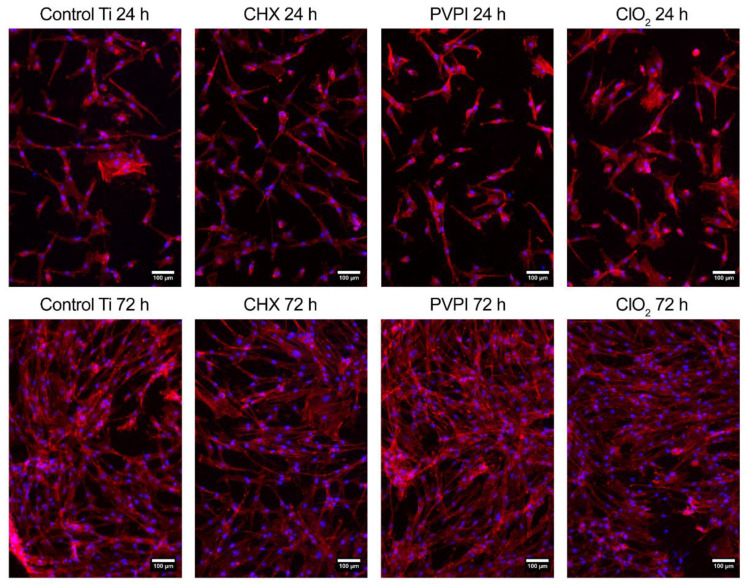
Fluorescent images of primary osteoblast cells’ attachment and proliferation after 24 and 72 h at a magnification of ×100.

Fluorescent images of primary osteoblast cells are presented in Figure 4. The actin cytoskeleton of the cells is shown in red (TRITC-phalloidin), and the cell nucleus is in blue (Hoechst 33342). No considerable difference from the controls was found in the attachment of osteoblast cells in any of the treated groups. After 72 h, we observed a considerable increase in cell numbers in each group, with cells covering the surfaces profusely.

**Figure 5:**
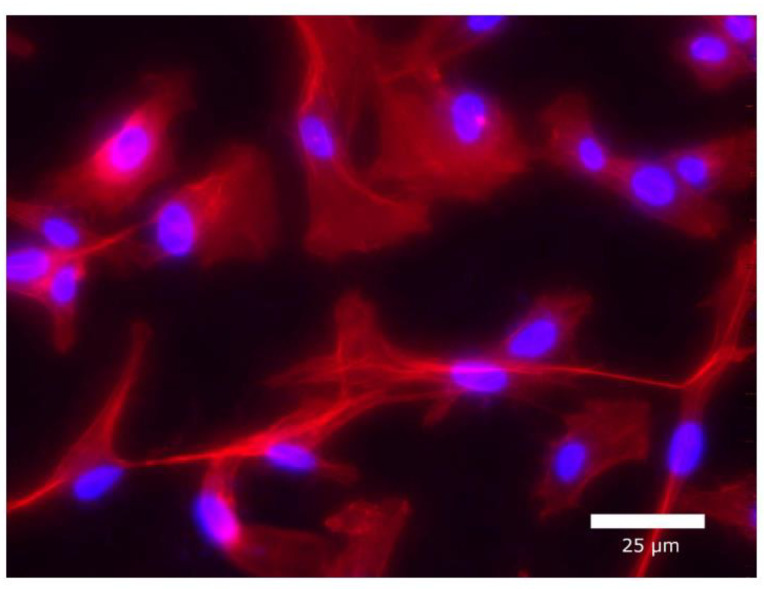
Fluorescent images of primary osteoblast cells on PVPI-treated discs after 24 h at a magnification of ×400.

Characteristic polygonal osteoblast cell morphology with some filopodia was detected in all investigated groups (Figure 5).

## 4. Discussion

In this study, we examined the surface chemical, compositional changes and cellular response on chemically treated titanium dental implant models. Surface hydrophilicity and SFE are important factors that could alter cell responses. Our CA experiments demonstrated that only povidone-iodine treatment could preserve a hydrophilicity similar to that of the control surface, while CHX and ClO_2_ treatment led to increased contact angle and decreased SFE values. In spite of this significant difference in wettability—but still in the hydrophilic range (Θ < 90°)—cell proliferation and biocompatibility tests showed similar cell populations in every treated sample. Generally, a significant change in SFE is considered to be a fundamental factor in terms of biological responses. Kubies et al. investigated osteoblast cell proliferation on different implant materials, in addition to contact angle and surface free energy measurements [30], finding that osteoblast cell proliferation was more pronounced on Ti surfaces with higher surface free energy. Rosales et al. also found that MG-63 cell adhesion was higher on sand-blasted, acid-etched surfaces with lower contact angles (higher hydrophilicity) [41]. These studies apparently contradict our results, but this difference could be explained by the fact that the CHX and ClO_2_ treatments changed the contact angles to a higher degree, but these remained still in the hydrophilic range (Θ between 39°–47°). Another reason can be the different response of primary oral human osteoblast cells compared with that of the MG-63 cell line.

Olvera-Huertas et al. studied the effect of CHX on the growth and differentiation capacity of primary human osteoblasts [42]. The study was not performed on Ti surfaces; direct 1 min treatment was applied with CHX (0.12% or 0.2%). Besides CHX, they investigated the effect of two antibiotics (amoxicillin and clindamycin). Alkaline phosphatase (ALP) activity was determined spectrophotometrically. Their data support the use of low doses of clindamycin and amoxicillin for intraoral bone graft decontamination and raise questions about the use of CHX as reduced cell proliferation and increased amount of apoptotic cells were found.

The three-time rinse in ultrapure water seems to be effective in the removal of antibacterial agents, since our XPS measurements indicated only the typical elements (Ti, O, and C) of titanium implant surfaces. CHX persistence in titanium surfaces was investigated earlier, where greater antibacterial activity was found with 0.2% CHX and rougher Ti surfaces [43]. Ungvári et al. also confirmed CHX remnants on Ti surfaces, although they investigated a more concentrated CHX gel [7]—in contrast to our study, where a 0.2% rinse was used.

To our knowledge, no studies before have been conducted involving cell cultures on titanium surfaces after povidone-iodine and chlorine dioxide treatment. This study showed that human osteoblast cells are capable of attaching and proliferating on these treated surfaces. This is a fundamental step in a possible re-osseointegration. Although in a real clinical situation, primarily epithelial and mucosal cells will be exposed to these solutions, in the case of regenerative therapy of peri-implantitis, osteoblasts could also face the titanium surface treated with chemical agents.

The antibacterial efficiency of these solutions was proven by our research group previously [44]. Venkei et al. found that 5 min treatment of PVPI and ClO_2_ led to significant elimination of *Streptococcus mitis* and *Streptococcus salivarius* in their in vitro study. Our next goal would be a collective study of the cellular viability of osteoblast cells on Ti surfaces previously coated with bacteria and treated with these antibacterial solutions.

Povidone-iodine has for a long time been a well-accepted antibacterial solution, while chlorine dioxide is a recently discovered antiseptic agent, especially in dental applications. Our results suggest that both of these agents could have an effective role as supplemental chemical therapy besides mechanical cleaning.

## 5. Conclusions

Taking all these results into consideration, CHX, PVPI, and ClO_2_ solutions are attested to be proper chemical decontamination agents supporting mechanical cleaning in the therapy of peri-implant diseases. Their antibacterial efficiency has been confirmed by several studies, and the present study has shown a favorable cellular response. We demonstrated that the chemical composition of the Ti surface was not altered and that primary oral osteoblast cells were not sensitive to the slight but significant difference in contact angle—as the cells could proliferate on each of the treated surfaces. Further in vitro and in vivo studies are required to better understand the response of other adjacent cells and tissues.

## Figures and Tables

**Table 1:** The SFE (γ) and its disperse (*γ*^d^) and polar (*γ*^p^) components of the discs, calculated with the Owen–Wendt–Rabel–Kaelble (OWRK) method. Data presented as means. Asterisks denote significant differences (* *p* <0.05, ** *p* < 0.01, and *p* < 0.001).

Surface	SFE γ (mJ/m^2^)	γ^d^ (mJ/m^2^)	γ^p^ (mJ/m^2^)
Control Ti	70.9	36.6	34.3
CHX treated	59.5 **	37.8	21.7
PVPI treated	70.7	36.5	34.2
ClO_2_ treated	64.1 *	37.8	26.4

## Data Availability

Not applicable.
